# Challenges and progress toward tumor-targeted therapy by systemic delivery of polymer-complexed oncolytic adenoviruses

**DOI:** 10.1038/s41417-022-00469-y

**Published:** 2022-04-20

**Authors:** Thavasyappan Thambi, JinWoo Hong, A-Rum Yoon, Chae-Ok Yun

**Affiliations:** 1 Department of Bioengineering, College of Engineering, Hanyang University, 222 Wangsimni-ro Seongdong-gu, Seoul, 04763 Korea; 2 GeneMedicine CO., Ltd., 222 Wangsimni-ro Seongdong-gu, Seoul, 04763 Seoul, Korea; 3 Hanyang Institute of Bioscience and Biotechnology (HY-IBB), Hanyang University, Seoul, 04763 Korea; 4 Institute of Nano Science and Technology (INST), Hanyang University, 222 Wangsimni-ro, Seongdong-gu, Seoul, 04763 Korea

**Keywords:** Targeted therapies, Nanoparticles

## Abstract

Oncolytic adenovirus (oAd) elicits antitumor activity by preferential viral replication in cancer cells. However, poor systemic administrability or suboptimal intratumoral retainment of the virus remains a major challenge toward maximizing the antitumor activity of oAd in a clinical environment. To surmount these issues, a variety of non-immunogenic polymers has been used to modify the surface of oAds chemically or physically. Complexation of oAd with polymers can effectively evade the host immune response and reduces nonspecific liver sequestration. The tumor-specific delivery of these complexes can be further improved upon by inclusion of tumor-targeting moieties on the surface. Therefore, modification of the Ad surface using polymers is viewed as a potential strategy to enhance the delivery of Ad via systemic administration. This review aims to provide a comprehensive overview of polymer-complexed Ads, their progress, and future challenges in cancer treatment.

## Introduction

Cancer is the second leading cause of death, responsible for loss of 10 million lives globally in 2020. Despite advancements in treatment options, including surgery, chemotherapy, and radiotherapy, the prognosis of patients with cancer remains poor because of the clinically elusive nature of the disease [1]. In addition, off-target effects in normal tissues remain a major safety concern [2, 3]. As conventional cancer treatment options remain insufficient, the development of new therapeutic approaches, such as oncolytic virotherapy, are under active investigation [4]. Unlike conventional therapies, oncolytic virotherapy uses replication-competent viruses that selectively infect, replicate, and induce lytic effects in cancer cells without harming normal cells [5].

Many oncolytic viruses (OV), including adenovirus (Ad), alphavirus, herpes simplex virus (HSV), poxvirus, Newcastle disease virus, poliovirus, measles virus, rhabdovirus, coxsackie virus, and vaccinia virus, are being actively investigated [6]. A genetically modified oncolytic Ad (oAd), H101, Oncorin®, in combination with chemotherapeutic drugs, was the first OV to be approved by the State Food and Drug Administration (SFDA) of China in 2005 [7]. To date, talimogene laherparepvec (T-Vec; Imlygic®), an oncolytic HSV expressing granulocyte-macrophage colony-stimulating factor (GM-CSF), remains the first and only virus to be approved by the United States Food and Drug Administration (FDA) in 2015 [8]. Approval of Imlygic has accelerated the clinical development of numerous OV pipelines. Currently, oAds are the most extensively investigated viruses in clinical development, owing to their excellent gene delivery efficacy, oncolytic effect, and facile production at high titers, as well as their ability to induce robust antitumor effects [9, 10]. However, nonspecific liver accumulation of systemically delivered Ad is especially problematic in the application of cancer therapy because a therapeutic that relies on local delivery cannot effectively treat distant metastases in the advanced stages of the disease [11]. Other factors like heparan sulfate proteoglycans (HSPGs) and coagulation factor IX also promote nonspecific hepatic sequestration of systemically delivered Ad [12, 13]. Therefore, the native tropism of systemically administered Ad limits therapeutic efficacy against disseminated disease and induces unwanted side effects like hepatotoxicity and thrombocytopenia [14].

In recent years, various strategies have been investigated to prevent rapid recognition and clearance of systemically administered Ad by the host immune system and its nonspecific sequestration into liver tissues [15]. One of the strategies explored most frequently has been masking the surface of Ad by coating with non-immunogenic polymers [16]. Masking the Ad capsid with polymers has been shown to attenuate nonspecific hepatic sequestration and prolong the blood retention time of systemically administered Ads [17–20]. Furthermore, the tumor-targeted strategy of systemically administered Ads facilitated tumor-specific delivery.

In this review, we highlight the advancements in polymer-complexed Ad delivery systems, aiming to explore how different types of polymers and targeting strategies are being used to overcome the limitations of conventional Ad. In addition, the review attempts to provide future directions in the design of efficient polymer-Ad vectors, briefly discussing the challenges toward the clinical development of polymer-Ad formulations, including biosafety and regulatory considerations, and how these challenges can be addressed.

## Chemical conjugation of Ads using polymers

To date, various polymers, including polyethylene glycol (PEG), poly-*N*-(2-hydroxypropyl methacrylamide) (pHPMA), and cationic polymers, have been used to mask the surface properties of Ads [21, 22]. Chemical conjugation of polymers to the Ad capsid enables the virus to escape detection by the host immune response system and suppresses nonspecific hepatic sequestration via systemic injection [16]. In most cases, chemical conjugation of polymers to the surface of Ad exploits the abundant lysine residues of Ad fiber proteins, and in general, conjugation of polymers to biomolecules reduces immunogenicity and interaction with blood proteins, which improves the blood circulation time and virus accumulation in target tissues [23].

### PEGylation of Ads

PEG is a hydrophilic and neutrally charged linear polymer well known for its biocompatibility and non-immunogenic properties [24]. Due to these attributes, conjugation of PEG to biomolecules has demonstrated increased hydrodynamic volumes and shielding of the molecule of interest, prolonging the half-lives of these PEGylated complexes in the bloodstream [25–27]. Similar to other PEGylated biomolecules, PEGylated Ads can evade detection by the host immune system under systemic circulation and have shown prolonged blood circulation time compared to naked Ad [28, 29].

PEGylation of Ad was first reported by O’Riordan et al. in 1999 [30]. Their findings showed that PEGylated Ad was more resistant toward inactivation by Ad-specific neutralizing antibodies (NABs) compared to naked Ad due to the effective shielding of capsid proteins, such as fiber, hexon, and penton base, by PEG. Cryole et al. also reported that PEGylation of Ad was a simple and practical approach to evade NABs [31]. Nevertheless, the application of these studies has been limited to intratracheal injections because of the aggregation and precipitation of functionalized Ads. Wilson et al. demonstrated that systemic administration of PEGylated Ad caused a lower level of adaptive immune response against Ad than the naked virions [32]. In detail, PEGylated Ad induced lower levels of Ad-specific NAB production against viral antigens than naked virion [32]. PEGylation of Ad has also been shown to attenuate induction of the innate antiviral immune response, evident by a lower level of interleukin (IL)-6 in serum following systemic administration compared with naked Ad [28, 33, 34]. While most studies have focused on PEGylation of human serotype 5 Ad, systemic administration of PEGylated human serotype 6 Ad has also been shown to induce lower levels of innate antiviral immune response and hepatotoxicity than the naked serotype 6 Ad [35]. These results illustrate that PEGylation of Ad could be considered a viable strategy to overcome the host immune response against systemically administered Ad.

The molecular weight of PEG and the degree of surface modification by PEG are key determinants in efficient PEGylation of Ad [36–38]. For instance, the surface modification of Ad with low-molecular weight PEGs (2–5 kDa) failed to reduce the accumulation of the virus in the liver [36]. Others have reported that, even with modification of 90% of virus surface lysine with 5 kDa PEG, systemic administration of PEGylated Ad failed to attenuate hepatic transgene expression compared with unmodified Ad [37]. Interestingly, covalent attachment of high-molecular weight PEG (20–35 kDa) to Ad significantly reduced liver accumulation [39]. Yao et al. [40] have reported 45% PEGylation (20 kDa) of reactive amines to be optimal PEGylation of Ad, as evidenced by significant reduction in the hepatic accumulation of Ad. This can be explained by the high molecular weight of PEG, which increases the size of the PEG-Ad complex and results in effective inhibition of PEG-Ad complex infiltration through liver sinusoids.

In general, PEGylation can ablate the native liver tropism of Ad by primarily blocking coxsackie and Ad receptor (CAR) interactions and increasing overall particle size to reduce sinusoidal infiltration [41]. Despite the advantageous attributes of PEGylation in systemic delivery of Ad, ablating the CAR binding capacity of Ad has been reported to reduce several critical aspects that are integral to its high transduction efficacy [42]. Ads enter cells through a two-step process. Firstly, the Ad fiber protein binds to CAR, which is subsequently assisted by affinity interaction between cellular integrins (α_*v*_β_*3*_ and α_*v*_β_*5*_) and arginine-glycine-aspartate (RGD) motifs located on the penton bases of Ad. In addition to the CAR, coagulation factor X specifically binds to the major capsid protein hexon contributes to native tropism of Ad [43]. Notably, PEGylated Ad demonstrates lower cellular internalization and microtubule-assisted trafficking of virions to the nucleus [23]. Therefore, more systematic studies are needed to address the shortcomings associated with PEGylated Ad.

### pHPMA conjugation of Ads

The pHPMA is another hydrophilic polymer that has been extensively used to conjugate with Ad. For instance, Fischer et al. conjugated pHPMA to the surface of Ad to achieve efficient liver detargeting and prevention of Ad neutralization by NABs [44]. In vitro studies demonstrated that 74% of the amine groups on the Ad had been modified by pHPMA. These modifications reduced Ad interactions with NABs by more than 80% [44]. Further, pHPMA-conjugated Ads were not taken up by A549 cells, indicating ablation of CAR-dependent endocytosis [44]. Additional studies were carried out to examine the in vivo fate of pHPMA-modified Ads [45]. Intravenous (i.v.) administration of pHPMA-modified Ads showed ~10,000-fold decrease in hepatic transgene expression level compared to naked virion, suggesting that pHPMA can prevent hepatic sequestration of i.v. administered Ad [46]. This effect is mainly due to the dense coverage of Ad capsid by pHPMA, effectively blocking coagulation factor X from binding with the Ad hexon [47].

In summary, pHPMA modification of Ad can effectively reduce the host immune response, ablate native liver tropism, and evade NAB-mediated neutralization. However, the presence of multiple reactive sites allows self-cross-linking reaction with Ad capsids, which reduces the transduction efficiency of Ad [48].

### Retargeting of PEG- or pHPMA-conjugated Ads

Tumors are known to express various receptors such as folate, integrin, HER2, endothelial growth factor (EGF), and fibroblast growth factor (FGF) receptors at a higher level than noncancerous tissues [49]. Therefore, various complementary tumor-targeting moieties that selectively bind to overexpressed receptors on the surface of different cancer cells have been actively investigated to improve tumor-specific accumulation of nanomaterials and their therapeutic cargos [50]. One of the most commonly explored adaptations of the strategy employs bifunctional PEG to conjugate both Ad and active tumor-targeting moieties, such as small molecule ligands, peptides, and antibodies [51].

For instance, in 2005, Eto et al. introduced an RGD peptide to a heterobifunctional PEG, which contains an amine group that reacts with Ad and maleimide to react with the thiol group of the c-terminal cysteine peptide residue of RGD [52]. Ad conjugated with PEG containing the RGD motif (RGD-PEG-Ad) exhibited good selectivity and strong affinity to various integrin-overexpressing cancer cells, showing up to 200-fold higher gene expression level in integrin-positive B16BL6 cells compared with PEGylated Ad and unmodified Ad. The competition assay performed by pre-treating the B16BL6 cells with RGD peptide revealed that the RGD-PEG-Ad particles were internalized specifically into the cells through integrin. Furthermore, i.v. administration of RGD-PEG-Ad in integrin *α*_v_*β*_3_-positive MDA-MB435 and U87MG tumor models led to a lower level of hepatic sequestration and a higher level of tumor accumulation with respect to naked Ad. This demonstrates that the active tumor-targeting moiety, RGD, on the surface of PEGylated Ad can improve tumor-specific systemic delivery of Ad.

Other groups have reported similar systemic delivery enhancements of Ad by conjugating bifunctional PEG to Ad and a tumor vasculature homing peptide (CGKRK; Ad-PEG_CGKRK_) [53]. Systemic administration of Ad-PEG_CGKRK_ particles to tumor-bearing mice promoted virus accumulation in tumor vessels and 500-fold higher transgene expression levels than naked Ad, and the complex induced 400-fold lower hepatic transgene expression levels compared to unmodified Ad [53]. Together, these studies demonstrate that the inclusion of a tumor-targeting moiety in PEGylated Ad formulations can improve tumor-specific accumulation and achieve liver de-targeting by systemic delivery.

Due to the presence of excessive extracellular matrix (ECM) in the desmoplastic pancreatic tumor microenvironment (TME), conventional therapies cannot effectively disperse within tumor tissues to induce a sufficient therapeutic effect [54]. Pancreatic cancers have been shown to express higher levels of neurotensin receptor type 1 (NTR) compared to normal tissues [55]. For this reason, NTR has been recognized as a promising candidate to enable targeted delivery to pancreatic tumors. Taking these factors into consideration, Na et al., have developed a pancreatic tumor-targeted nanohybrid vector that combines an ECM-degrading and Wnt/β-catenin signal inhibiting oAd with NTR targeting neurotensin peptide (NT)-conjugated PEG (oAd/DCN/LRP-PEG-NT) [56]. The oAd/DCN/LRP co-expresses decorin (DCN), which is a leucine-rich proteoglycan that suppresses the production of ECM components by inhibiting the activity of TGF-β, and soluble Wnt decoy receptor (LRP), which prevents the Wnt/β-catenin signaling pathway from promoting epithelial-to-mesenchymal transition (EMT) and metastasis of the tumor [57–59]. Systemic administration of oAd/DCN/LRP-PEG-NT (tumor volume: 98 ± 9.2 mm^3^) showed a 2.3-fold greater antitumor effect when compared with naked oAd/DCN/LRP (tumor volume: 221.3 ± 26.9 mm^3^) in pancreatic tumor tissue [56]. Interestingly, the distribution of collagen fibers, a major component of ECM, in tumor tissue was decreased by oAd/DCN/LRP-PEG-NT. These results demonstrate that oAd/DCN/LRP-PEG-NT can be delivered efficiently to pancreatic tumor tissues after systemic injection where it induces substantial expression of DCN, resulting in enhanced antitumor efficacy through downregulation of EMT and Wnt-related factor.

In another study, Kim et al. examined the possibility of treating Her2/neu-positive cancer using Herceptin-conjugated and PEGylated Ad (DWP418-PEG-HER) [60]. This study examines the in vivo efficacy of DWP418-PEG-HER for the treatment of ovarian and breast cancer. The Her2/neu-targeted Ad effectively transduced Her2/neu-overexpressing cancer cells via a specific interaction between Herceptin immobilized on the surface of PEGylated oAd and Her2/neu expressed on the surface of cancer cells. DWP418-PEG-HER conjugate showed longer circulation time and lower induction of NABs and IL-6 than naked oAd, which suggested that PEG conjugation reduces the immune response and prolongs oAd blood circulation [60]. The attributes of DWP418-PEG-HER can protect the virus after systemic injection, which subsequently influences in vivo therapeutic efficacy in Her2/neu-positive tumor models (SKOV-3 and MDA-MB435). DWP418-PEG-HER induced 4.0- and 4.3-fold higher antitumor activity than nontargeted PEGylated Ad (DWP418-PEG) and naked Ad in an SK-OV3 tumor model, respectively. More importantly, DWP418-PEG-HER showed 3.7 × 10^3^-fold higher tumor accumulation and 2.8 × 10^6^-fold lower liver sequestration compared with naked Ad. Ultimately, this led to a 1.3 × 10^10^-fold increased tumor-to-liver ratio in the MDA-MB435 tumor model. In contrast, Her2/neu-negative MCF7 tumor growth was not regressed, and the antitumor effect of DWP418-PEG-HER was similar to that of DWP418-PEG. Together, these results suggests that DWP418-PEG-HER, which exhibits HER2-targeting ability, potent therapeutic efficacy, and safety, is a promising therapeutic for systemic treatment of HER2-overexpressing cancer.

In addition to PEG, pHPMA-conjugated Ad was labeled with FGF or VEGF to ablate native tropism and improve tumor targetability [44]. Unlike monofunctional PEG which contains only one reactive group, pHPMA possesses multiple reactive groups that are known to bind to the surface of particles, resulting in lateral and steric stabilization against disruption from biological molecules [61]. The presence of multiple reactive groups allows pHPMA to be conjugated with other functional moieties, such as drugs or targeting ligands [62]. For example, Fisher et al. introduced FGF or VEGF onto pHPMA-coated Ad [44]. The polymer-coated Ad with targeting ligands exhibited ligand-dependent and CAR-independent cell uptake, as confirmed by a competition assay using FGF or VEGF antibodies. Other studies have demonstrated that growth factors, such as EGF, can also be used as a tumor-targeting moiety on the surface of pHPMA to redirect the Ad internalization of EGFR-positive cells. Morrison et al. introduced cetuximab, an EGFR-targeted antibody, on pHPMA to retarget Ad to EGFR-positive cancer cells [63]. The modified Ad particle exhibited enhanced transgene expression in EGFR-positive cells (T24 and A431 cells) but did not increase transgene expression in EGFR-negative cells (SW620 cells), demonstrating EGFR-specific targeting. In vivo antitumor effect of EGFR-targeted pHPMA modified Ad (Cx-p-Ad) was examined by imaging the bioluminescence of intraperitoneally injected SKOV-3-luc cells in MF1 nude mice. After 10 days, mice treated with Cx-p-Ad exhibited significantly reduced bioluminescence when compared with mice treated PBS or naked Ad.

Although intriguing results have been obtained by these studies, the use of growth factor-based targeting ligands limits the tumor-targeting ability of Ad because of the random conjugate formation or lack of site-specificity leads to heterogeneous products formation [64, 65]. Furthermore, some growth factor-based ligands possess cationic domains that induce unwanted interaction with erythrocytes during systemic administration [66]. Importantly, growth factor-based targeting ligands possess several reactive functional groups, which not only limit their site-specific conjugation with polymers, but also form cross-linking reactions with Ad and reduce its infectivity [23]. Therefore, these factors must be considered when developing targeting ligand-conjugated Ad/polymer complexes.

### Cationic polymer-conjugated Ads

Cationic polymers are attractive viral gene delivery carriers. Cationic polymers form a nanosized polyplex with genetic materials by ionic complexation [67, 68]. Several viral vectors have been complexed with cationic polymers to increase their gene transfer efficacy [69]. After complexation, the surface charge of therapeutics is converted from negative to positive, increasing charge interaction with the anionic cell membrane to enhance the cellular uptake of therapeutic cargo [69]. For instance, Ads complexed with various cationic polymers, including polylysine, polyethylenimine (PEI), polyamidoamine dendrimer (PAMAM), poly(aminoester), and chitosan, have been shown to induce a higher level of transgene expression than the naked Ad [70]. Conjugation of cationic polymers to Ad was conducted by Kim et al., who modified the surface of Ad using an arginine-grafted biodegradable polymer (ABP) [71]. In this study, hepatocellular carcinoma (HCC)-selective Ad (YKL-1001) was conjugated using ABP to obtain a YKL-1001-ABP complex. The YKL-1001-ABP complex elicited more potent cell-killing effects in Huh7 and HepG2 liver cancer cells when compared with naked YKL-1001. Interestingly, YKL-1001-ABP induced negligible cell killing effects against non-HCC cancer cells (A549, PC-3, and HT1080 cells), suggesting that the HCC-specific anticancer effects of YKL-1001 were retained after surface modification with ABP. Furthermore, mice treated with YKL-1001-ABP exhibited 7-fold lower proinflammatory IL-6 cytokine secretion compared with naked YKL-1001, demonstrating efficient attenuation in induction of the innate antiviral immune response. Systemic administration of the YKL-1001-ABP complex resulted in no significant increase in the serum AST and ALT levels, indicating an absolute reduction in liver toxicity, whereas mice that received naked YKL-1001 showed hepatocellular swelling and nuclei degeneration. Owing to these properties, i.v. administration of the YKL-1001-ABP complex induced more potent tumor growth inhibition of the Huh7 HCC xenograft tumor model than naked YKL-1001. In summary, the chemical conjugation of cationic polymers or other hydrophilic polymers (e.g., PEG and pHPMA) reduce the hepatic sequestration and enhances antitumor efficacy.

## Physical coating of Ads using polymers

### Cationic polymer-coated Ads

Cationic polymers have been used widely to coat the anionic surface of Ads to generate a complex with a net cationic charge that can internalize into a wide range of cancer cells in a CAR-independent manner [69]. CAR-independent internalization of Ad is critical to maximizing the therapeutic potential of Ad in a clinical environment where heterogenic tumor populations exhibit variable CAR expression or abrogation of CAR altogether [72]. A series of pHPMA with different lengths of pendant cationic oligolysines (pHPMA-*co*-oligolysine; pHK_5_, pHK_10_, and pHK_15_) was synthesized to coat Ad5 by charge interaction [73]. The transduction of Ad5 coated with different oligolysine chain lengths containing pHK variants revealed that pHK_10_ showed better transduction than the other two formulations. The transduction efficiency of Ad5 complexed with pHK_10_ was significantly increased (sixfold) in several cells (CHO-K1, RAW264.7, and BaF3 cells) that lack CAR expression in comparison to unmodified Ad5. Importantly, pHK_10_-coated Ad5 retained its transduction even in the presence of high serum levels of Ad-specific NABs, which indicated complete shielding of Ad by the HPMA-*co*-oligolysine formulation. Furthermore, a transduction efficiency test in CAR-defective CHO cells demonstrated greater transduction than that of unmodified Ad5, suggesting that the cationic pHK_10_ formulation overcomes CAR-dependent cellular entry of Ad because of pendant oligolysine in the HPMA provided high-affinity virus binding assisted by the polyvalent characteristics of pHK. Similarly, Francini et al. developed a cationic pHPMA with diazonium functional groups at different densities (85, 165, and 240) to coat the oncolytic Ad enadenotucirev (EnAd) [74]. Polymer coating of EnAd using pHPMA containing a high degree of polyvalent diazonium groups (i.e., 240) efficiently shielded viral capsids and effectively ablated NAB binding to the Ad surface. The transduction efficiency of polymer-coated EnAd was initially lower but slowly increased as the time progressed, suggesting that Ad could be slowly released from the polymer coating with a longer incubation time. Thus, the introduction of cationic functional groups or oligomers in hydrophilic pHPMA can be used as a suitable strategy to coat Ad for the effective infection of cancer cells.

To further examine the role of cationic polymers in promoting Ad internalization in cancer cells, Choi et al. developed a series of six biocompatible polymers based on PEG-poly(*N*-[*N*-(2-aminoethyl)-2-aminoethyl]-L-glutamate) (PEG-PNLG) copolymers with different PEG molecular weights (2, 3.4, or 5 kDa) and numbers of amine groups (2 or 5) [75]. PEG-PNLG copolymers effectively coat Ad vectors and form positively charged stable nanoparticles. The size and surface charges are relatively proportional to the amine numbers. For example, Ad complexed with a PEG-PNLG variant containing five amine groups exhibited both larger size and more cationic charge (210 nm and 48 mV, respectively) than a complex generated with a polymer variant with a lower amine density of two (120 nm and 33 mV, respectively). Furthermore, the Ad nanocomplex prepared with the PEG-PNLG variant containing five amine groups exhibited higher transduction efficacy than Ad complexed with PEG-PLNG containing two amine groups in both CAR-positive and CAR-negative cells, indicating that the greater number of amine groups in the polymer is integral to improving the in vitro transduction ability of Ad [75].

Lee et al. developed Ad-coated with a cationic polymer, deoxycholic acid-conjugated poly(ethyleneimine) (DA3), to enhance Ad delivery into cancer cells with low CAR expression [76]. The Ad/DA3 complex enhanced gene transfer efficiency in CAR-negative HT1080 cells, and was internalized by clathrin-, caveolae-, and micropinocytosis-mediated endocytosis. Furthermore, an antiangiogenic oAd (RdB-KOX that expresses VEGF-targeted artificial transcriptional repressor zinc-finger protein) complexed with DA3 (RdB-KOX/DA3) has been shown to elicit a more potent antiangiogenic and cytotoxic effect than naked RdB-KOX [76]. The enhanced cancer cell death effect of the RdB-KOX/DA3 complex was translated in vivo. The antitumor effect of the RdB-KOX/DA3 complex was improved dramatically in a human tumor xenograft model showing 94.34% tumor inhibition compared with the 59.1 1% inhibition of naked RdB-KOX. Taken together, these findings suggest that cationic polymer-complexed Ad can overcome CAR dependency in cancer cells (Table 1).

**Table 1:** Polymer-coated Ads for cancer therapy.

Oncolytic Ad	Polymer	Targeting ability	Therapeutic efficacy	X^a^	Ref.
Ad-GFP	pHPMA-*co*-oligolysine	Passive targeting	Increased transduction in cells that lack CAR	–	[73]
RdB-KOX	DA3 (*b*PEI-*g*-DOCA)	Passive targeting	Enhanced apoptosis, reduced proliferation and angiogenesis, and increased viral production	–	[76]
dAd-GFP	PPE (ErbB-conjugated PEGylated PAMAM)	Selective internalization to EGFR (+) cells	Prolonged blood retention and enhanced tumoral accumulation, and potent therapeutic effect in lung tumors	i.v.	[92]
Ad-∆E1-GFP	Chitosan-PEG-FA	Specificity against FA receptors	Enhanced transduction and tumor regression in FA-positive tumors	i.v.	[90, 91]
RdB-KOX	PEG-*b-*PHF	Passive targeting	Lower hepatic toxicity and no induction of immune response, and enhanced antitumor efficacy	i.v.	[87]

*i.v*. intravenous.

^a^Route of administration.

### Bioreducible cationic polymer-coated Ads

Numerous cationic polymers such as PEI, poly(lysine), and chitosan and liposomes have been used to complex with various gene therapeutics to enhance their gene delivery efficiency into a wide range of cell lines [77]. Among these candidates, 25 kDa PEI has long been the gold standard for gene delivery due to its excellent cell uptake and endosomal escape ability through a proton sponge effect [78]. However, 25 kDa PEI has been shown to be highly cytotoxic, and this limits its application in clinics [79–81]. To surmount the cytotoxicity issue and improve the gene transfer efficiency of PEI, bioreducible disulfide bonds-containing PEIs have been developed. These materials show superior gene transfer efficiency and lower cytotoxicity compared with non-degradable PEIs.

For instance, Choi et al. developed a multi-degradable, bioreducible, and core-cross-linked PEI (rPEI) copolymer of varying molecular weights (16 and 32 kDa) as non-toxic and non-immunogenic material to coat the surface of oAd [82]. In both CAR-positive and CAR-negative cells, Ad complexed with 16 kDa rPEI showed higher transduction efficiency and more potent cancer cell killing effect than naked Ad, Ad complexed with standard 25 kDa branched PEI, or Ad complexed with 32 kDa rPEI [82]. In another study, Jung et al. developed a multi-degradable cationic vector based on an mPEG-PEI-*g*-Arg-S-S-Arg-*g*-PEI-mPEG copolymer (PPSA) to coat Ad (Ad/PPSA) [83]. Physicochemical characterization of Ad/PPSA demonstrated the formation of a positively charged stable particle, <200 nm in diameter [83]. The Ad/PPSA nanocomplex exhibited enhanced transduction efficiency in both CAR-positive (A549 cells) and CAR-negative (MCF7) cells [83]. An oAd (DWP418) complexed with PPSA (DWP418/PPSA) elicited 2.24-fold greater antitumor effect in MCF7 tumor xenograft than uncoated DWP418 [83].

Similarly, biodegradable poly(disulfide amine) polymers with defined structures were synthesized using a Michael addition reaction between cystamine bisacrylamide and diamines [84]. To improve tumor-targeting characteristics of bioreducible polymer-coated oAds, they were loaded into bone-marrow-derived mesenchymal stromal cells (hMSCs) with tropism toward tumors. Unlike naked oAds that show poor loading into hMSCs, the poly(disulfide amine) (poly(CBA-DAH); PCDP)-coated oAds were loaded efficiently into hMSCs [85]. Systemic administration of oAd/RLX-PCDP-loaded hMSCs showed efficient accumulation and replication of oAd at tumor tissues, resulting in a 4.8-fold higher antitumor effect than that of the naked oAd/RLX group in an orthotopic pancreatic tumor model through enhanced induction of apoptotic tumor cell death and degradation of tumor ECM.

Regardless of the bioreducible polymer types, all bioreducible polymers showed reduced toxicity in comparison with conventional 25 kDa PEI. The introduction of poly(disulfides) into the polymers allows rapid degradation of the polymer in the intracellular environment, which contains high concentration of glutathiones that reduce disulfide bonds and release of oAd from the polymer coating (Table 2).

**Table 2:** Bioreducible cationic polymer coated Ads for cancer therapy.

Oncolytic Ad	Polymer	Targeting ability	Therapeutic efficacy	X^a^	Ref.
Ad-∆E1	ABP	Passive targeting	Enhanced transduction in both CAR-(+/−) cells	i.v.	[71]
RdB/shVEGF	PPCBA	Passive targeting	Enhanced cellular uptake in low or high CAR-expressing cells	i.v.	[89]
RdB/shMet	rPEI	Specificity against FA receptor	Enhanced cancer cell killing and increased viral production in CAR-(+/−) cells	–	[82]
DWP418	PPSA	Passive targeting	Enhanced transduction in both CAR-(+/−) cells	–	[83]
oAd/RLX	PCDP	Passive targeting	Potent antitumor effect and superior viral replication in pancreatic tumor model	i.v.	[85]
Ad-DB7-U6shIL8	CD-PEG_m_-RGD	Specificity against *α*_v_*β*_3_ and *α*_v_*β*_5_ integrin receptors	Enhanced cytopathic effect in integrin positive receptors	–	[94]

*i.v*. intravenous.

^a^Route of administration.

### TME-targeted and pH-responsive cationic polymer-coated Ads

Preferential tumor accumulation of systemically administered oAds can be enhanced by complexation with a polymer-containing targeting moiety. However, its high specificity toward a single target limits its efficacy in a clinical setting because tumor cells are heterogeneous. In short, targeting moiety-conjugated polymer cannot deliver oAd to cancer cells lacking complementary cellular receptors. To surmount this issue, TME-responsive polymers that target tumor-specific conditions have been utilized for complexation with Ad.

A pH-responsive moiety often is used to enhance the tumor-targeting ability of the polymer because TME is mildly acidic where normal physiological pH is close to neutral [86]. A pH-sensitive diblock copolymer PEG-*b*-poly(L-histidine-*co*-L-phenylalanine) (PEG-*b-*PHF) was developed to targeting acidic TME and inhibit tumor growth in a U87 tumor xenograft-bearing mice models [87]. The tumor-targeting property of the pH-sensitive polymer was examined by complexing it with an oAd-expressing transcription repressor of the VEGF promoter (KOX). Cancer cell migration and VEGF gene expression were significantly suppressed when the KOX/PEG-*b-*PHF complex was incubated at pH 6.4 compared to the complex incubated at pH 7.4, demonstrating the pH-responsiveness of the complex. Moreover, KOX/PEG-*b-*PHF (pH 6.4) elicited an enhanced cancer cell killing effect compared to both naked KOX and KOX/PEG-*b-*PHF (pH 7.4). Consequently, the systemically administered KOX/PEG-*b-*PHF complex induced more potent antitumor activity than naked KOX in the U87 tumor xenografts established in mice [87]. Inspired by this approach, the pH-sensitive property of the copolymers was further tuned by synthesizing a PEG-*b*-poly(L-histidine) (PEG-*b-*pHis) copolymer [88]. The Ad coated with PEG-*b-*pHis copolymer was protonated in the acidic pH conditions of tumor tissues and effectively covered the Ad to generate a PEG-*b-*pHis/Ad complex. In comparison with naked Ad, the transduction efficiency of the PEG-*b-*pHis/Ad complex at pH 6.4 was increased by 3-, 9-, and 339-fold in U343, A549, and MCF7 cells, respectively. The therapeutic efficacy of the mPEG-*b*-pHis/Ad complex was significantly enhanced in both CAR-positive (C33A) and CAR-negative (MCF7) tumor models, showing 57 and 49% tumor growth inhibition in comparison with naked Ad, respectively. Collectively, these results demonstrate that PEG-*b*-pHis-mediated delivery of Ad is greatly augmented by acidic TME.

The introduction of bioreducible linkers provides greater pH sensitivity and improves the release of coated Ad in the intracellular compartment of cancer cells. For instance, Moon et al. synthesized a pH-responsive and bioreducible copolymer (PPCBA) to coat the surface of oAd [89]. Owing to its pH responsiveness, the Ad-PPCBA complex showed enhanced cellular uptake at pH 6.0 compared to pH 7.4 in both CAR-positive and -negative cancer cells. Endocytosis pathway analysis demonstrated that, instead of the CAR-dependent endocytic pathway, internalization of the Ad-PPCBA complex was mediated by macropinocytosis. Importantly, intratumorally and i.v. administered VEGF-specific shRNA-expressing oAd-PPCBA nanocomplex induced a 3-fold more potent antitumor effect than naked Ad in human xenograft tumor models with a lower level of systemic toxicity [89]. Together, these results demonstrated that pH-sensitive nanomaterials can improve the tumor-specific systemic delivery of oAd.

### Active tumor-targeted cationic polymer-coated Ad

Other than passive targeting, introducing tumor-targeting moieties such as ligands and antibodies can actively target various receptors overexpressed on tumor tissues. For instance, Park et al. developed an folic acid (FA) anchored Ad/chitosan-PEG-FA nanocomplex to enable active tumor-targeting and tumor-specific transduction [90]. Increasing the feed ratio of FA in the nanocomplexes enhanced the transduction efficiency in FA-positive KB cells, whereas there was no significant influence on transduction efficiency of FA-negative U343 cells [90]. Infection of RAW264.7 macrophages using Ad/chitosan-PEG-FA nanocomplexes produced a lower level of inflammatory cytokine IL-6 compared to naked Ad [90]. Based on the outstanding in vitro properties of Ad/chitosan-PEG-FA nanocomplexes, further studies on Ad/chitosan-PEG-FA nanocomplexes were conducted to examine their antitumor efficacy in a subcutaneous KB tumor model [91]. Presence of PEG in the cationic nanocomplexes increased the blood circulation time to 49-fold higher than that of naked Ad at 24 h after systemic administration. Mice treated with Ad/chitosan-PEG-FA nanocomplexes showed 75.3% reduction in Ad-specific NAB generation compared with naked Ads and, more importantly, a 378-fold reduction in liver accumulation compared to that of naked Ad [91]. Moreover, systemic administration of Ad/chitosan-PEG-FA nanocomplexes to FR-positive KB subcutaneous tumor-bearing mice led to a 285-fold increase in intratumor virus accumulation compared with that of naked Ad. Owing to the enhanced tumor targeting of the Ad/chitosan-PEG-FA nanocomplex, systemic administration of the nanocomplex markedly inhibited the growth of FR-positive tumors. This was the first study to show effective tumor targeting of oAd that was systemically injected by physical coating with active tumor-targeting polymers.

In addition to natural cationic polymers, dendritic polymers with a well-defined structure, size, and surface charge have been explored in gene delivery. Poly(amidoamine) (PAMAM) dendrimers are highly water-soluble and non-immunogenic, and the presence of surface functional groups allows easy modification with drugs and targeting ligands. Yoon *et al*. used an EGFR-specific antibody, Erbitux-conjugated and PEGylated PAMAM dendrimer (PPE), to complex with Ad [92]. EGFR-targeted PPE-coated Ad specifically internalized into EGFR-positive A549 cells with twofold and sevenfold greater transduction efficiency compared to Ad coated with a nontargeted polymer (PP) or naked Ad, respectively. Importantly, systemic administration of oAd complexed with PPE (oAd/DCN-shMet/PPE) showed a higher level of virion accumulation in tumor tissues compared with that of naked Ad or PP-coated Ad (oAd/DCN-shMet/PP), demonstrating that the Erbitux on the surface of the PPE can enable efficient targeting of tumor tissues. Notably, the tumor-to-liver ratio of oAd/DCN-shMet/PPE was 1.7 × 10^9^-fold greater than that of naked oAd/DCN-shMet, implying increased tumor accumulation and decreased liver sequestration of oAd/DCN-shMet/PPE. Consequently, systemically delivered oAd/DCN-shMet/PPE showed a 14.9-fold higher level of tumor growth inhibition in an EGFR-positive orthotopic lung tumor model in comparison with naked Ad, ultimately resulting in complete tumor regression in 66% of the treated mice. The pharmacokinetic profile of the oAd/DCN-shMet/PPE complex demonstrated a 290-fold higher viral genome in the blood circulation after 24 h, which implied that effective masking of Ad reduces immunogenicity during circulation and enhances Ad circulation time for tumor targeting. This was the first report demonstrating that the oAd nanocomplex can effectively treat an orthotopic xenograft tumor that closely emulates the disease progression and properties of a clinical tumor [93]. Together, these studies illustrate that cationic nanocomplexes with tumor-targeting moieties can improve tumor-targeting properties of the complex in comparison to nontargeted materials.

Coating of Ad using cationic polymers provides numerous advantages including attenuation of the innate immune response, protection against blood proteins, prolonged circulation in the bloodstream, and increased targeting ability. The safety of cationic polymers can be further improved by the introduction of degradable linkers that can facilitate biodegradation and clearance of the polymers upon cellular internalization. For instance, to redirect cationic polymers containing multiple degradation linkers to tumor tissues, poly(cystaminebisacrylamide-diaminohexane) (CD) copolymer has been modified with a cyclic RGD (cRGDfC) to selectively target the tumor vasculature [94]. Ad complexed with cyclic RGD-conjugated CD (Ad/CD-PEG-RGD) in RGD showed strong induction of apoptosis and suppression of IL-8 and VEGF expression in HT1080 cells. More importantly, Ad/CD-PEG-RGD conjugate showed an oncolytic effect in both CAR-positive and CAR-negative cancer cells [94].

**Fig. 1:**
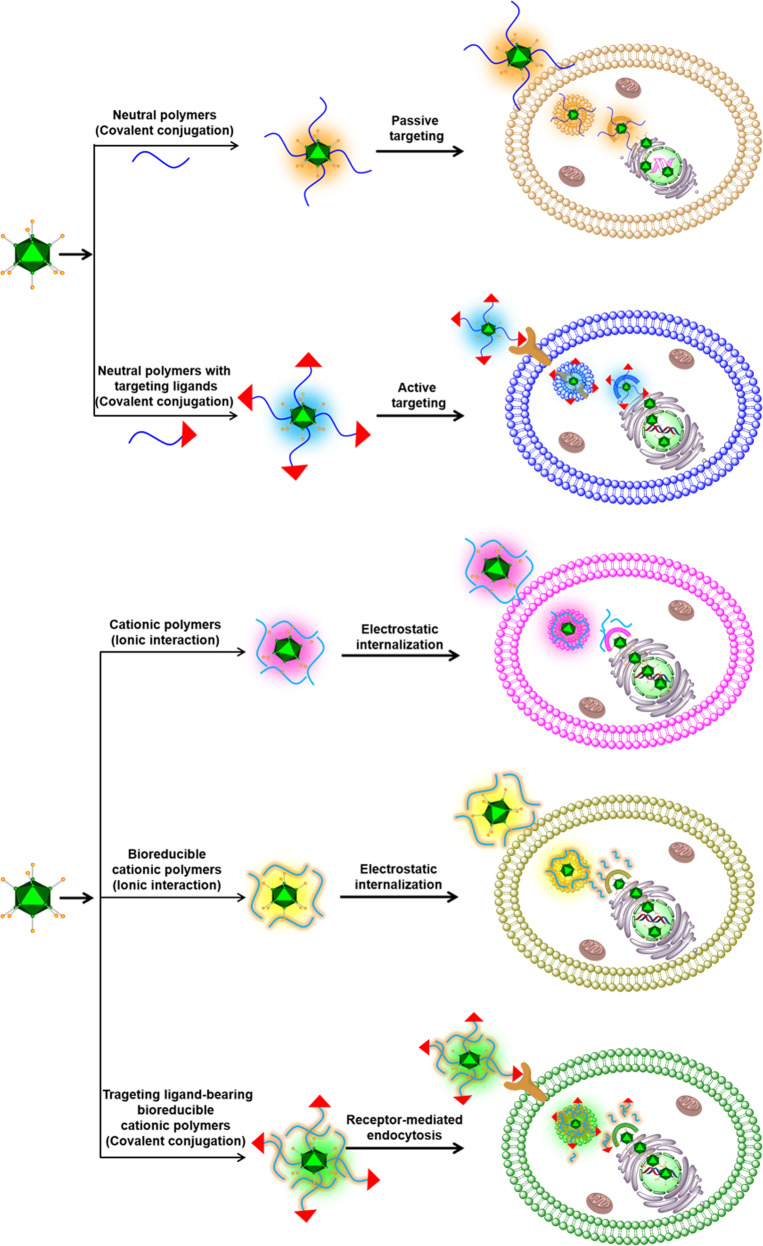
Current methodologies to modify oAd using polymer-coating or chemical conjugation. Modification of oAd using polymers enables the resulting complex to evade detection by the host immune system, thus achieving prolonged blood circulation time. Conjugation of hydrophilic and neutrally charged polymers (e.g., PEG and pHPMA) to the surface of oAds offers efficient protection against neutralization by Ad-specific NABs. The inclusion of tumor-targeting moiety on the surface of the nanocomplex further improves tumor-specific transportation of oAd by systemic administration. The surface of oAds can be coated or conjugated with cationic polymers (with or without bioreducible bonds) to increases the cellular uptake of oAds in CAR-deficient cells via electrostatic interaction. The presence of bioreducible bonds in the cationic polymers facilitates the release of oAds at the intracellular compartments or cytoplasm.

**Fig. 2:**
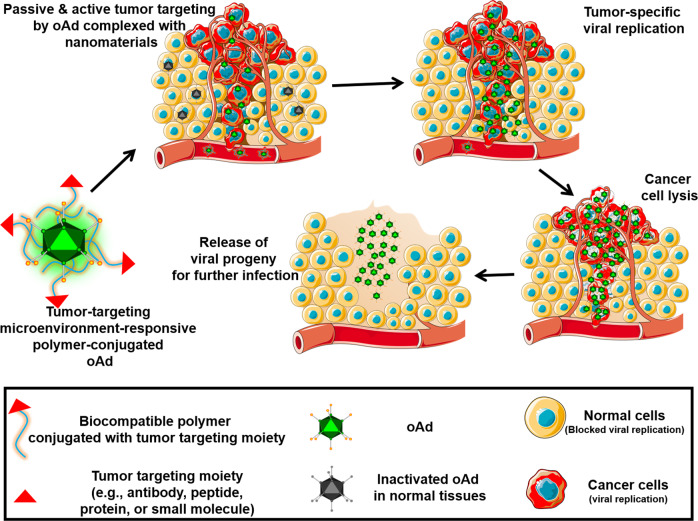
Systemic administration of oAds coated or conjugated with bioreducible cationic polymer containing tumor-targeting moiety. The oAds coated or chemically conjugated with tumor-targeted polymers internalize into the tumor tissues by binding to complementary receptors expressed on tumor cells (active tumor targeting). In addition, nanosized oAd complexed with the polymer can preferentially infiltrate into the leaky vessels of tumor tissues and be retained at a higher level than in normal tissues due to enhanced permeability and retention effect (passive tumor targeting). The harsh tumor microenvironment, such as low pH, upregulated redox potential, and hypoxia, triggers the release of oAds from polymer shielding upon internalization into tumor cells. As the exponential replication of oAds occurs in a highly tumor-specific manner, minimal off-target toxicity is observed even if some of the viral particles internalize into normal tissues via systemic delivery.

**Fig. 3:**
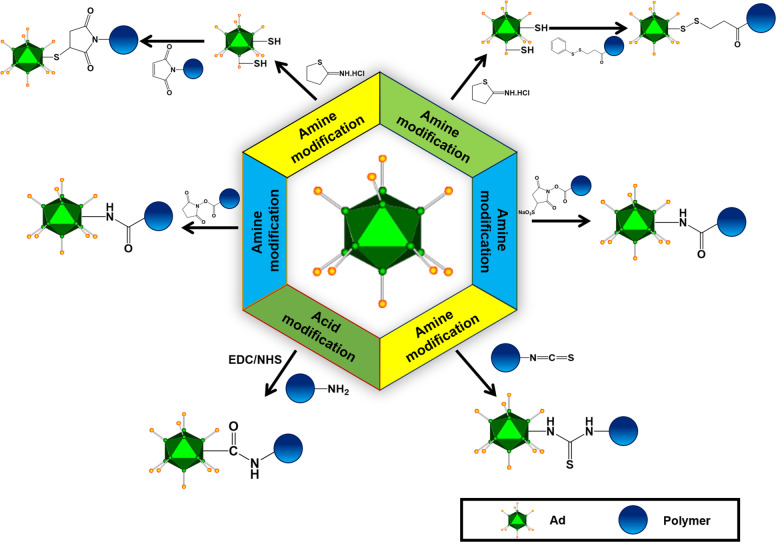
Chemical methodologies used for the modifications of Ads. The amine and carboxylic acid groups on the surface of Ad capsids are often used to directly conjugate polymers.

In summary, Ad complexed with cationic polymers conjugated with active tumor targeting moieties can enhance the antitumor activity of Ad via systemic administration (Figs. 1 and 2). Furthermore, the polymer-coating on the surface of Ads can attenuate the induction of an immune response against Ad and prolong the blood circulation time of the virus, implying that this strategy enables effective targeting of disseminated metastatic tumors. Various chemical methodologies used for the modifications of Ads are briefly summary in Fig. 3. The summary of polymer complexed or coated Ads and their antitumor effect was summarized in Table 3.

**Table 3:** Polymer complexed or coated Ads and their summary of antitumor effect.

Polymer (Formulation name)	Targeting ability	Antitumor efficacy^a^	Advantage	Disadvantage	Ref.
PEG conjugated Ad (DWP418-PEG-Ad)	Specific targeting to Her2/neu-positive receptor	(i) 4.3-fold higher for SKOV-3 tumor (ii) 2.04-fold higher for MDA-MB-231 tumor	Simple modification procedure	Limited targeting against Her2/neu-negative tumor	[60]
PPSA polymer-coated Ad (DWP418/PPSA)	Passive targeting	2.68-fold higher for MCF7 tumor	Good antitumor effect in Her2/neu-negative tumor	Multistep synthesis of PPSA	[83]
DA3 polymer-coated Ad (RdB-KOX/DA3)	Passive targeting	7.24-fold higher for HT1080 tumor	Enhanced antitumor effect in low CAR expressing tumors	Non-degradability of PEI polymer	[76]
PEG-*b-*PHF polymer coated Ad (KOX/PEG-*b-*PHF)	Passive targeting	2.95-fold higher for U87 tumor	Tumor acidic pH-sensitivity and biodegradation of copolymers into non-toxic fragments	Accumulation of Ad in liver	[87]
ABP polymer conjugated Ad (YKL-1001-ABP)	Specific targeting to hepatocellular carcinoma	2.48-fold higher for Huh7 hepatoma tumor	ABP polymers are sensitive to both pH and GSH and trigger the release of Ad in tumor cells	Controlling the molecular weight of ABP is difficult	[71]
Chitosan coated Ad (Ad/chitosan-PEG-FA)	Specific targeting to folate receptor	2.34-fold higher for KB tumor	Able to control the FA amount in the polymer for active targeting	Liver accumulation of the coated Ad	[90, 91]

^a^In comparision to naked or free Ad group.

## Conclusion and future perspectives

To maximize the therapeutic potential of oAds in a clinical setting, major limitations, such as poor systemic administrability and intratumoral retainment of the virus must be addressed. The development of a polymer-based carrier system for oAd is one of the promising approaches to address these inherent limitations of oAd, as these materials can endow novel properties such as low immunogenicity and improve the pharmacokinetics and tumor-targeting ability that are either impossible or difficult to achieve via conventional genetic engineering methods. Masking the oAd capsid using non-immunogenic polymers either by chemical conjugation or physical coating can prolong the circulation time of oAd in the bloodstream, attenuate induction of an antiviral immune response, and curtail nonspecific liver sequestration. Furthermore, inclusion of active tumor-targeting moieties can lead to tumor-specific accumulation of systemically administered oAd complex, simultaneously improving both the therapeutic efficacy and safety profile.

Despite remarkable advancement within the field of polymer-based delivery of oAds in the last decades, many aspects that must be carefully investigated prior to its transition into a clinical environment. For example, scale-up studies, improvement of the homogeneity of the complex, optimization of Ad complex generation procedures in a good manufacturing practice (GMP) environment, and more in-depth toxicology studies must be conducted. To this end, new polymerization techniques that can synthesize polymers with defined structures will be useful for bulk synthesis. Therefore, the development of tailored polymers could collectively overcome the limitations oAds and improve tumor-specific accumulation via passive and active targeting to achieve enhanced gene therapy for cancer.

## Data Availability

The data that support the findings of this study are available from the corresponding authors, upon reasonable request.
